# Requirement for Jagged1-Notch2 signaling in patterning the bones of the mouse and human middle ear

**DOI:** 10.1038/s41598-017-02574-7

**Published:** 2017-05-31

**Authors:** Camilla S. Teng, Hai-Yun Yen, Lindsey Barske, Bea Smith, Juan Llamas, Neil Segil, John Go, Pedro A. Sanchez-Lara, Robert E. Maxson, J. Gage Crump

**Affiliations:** 1 Eli and Edythe Broad CIRM Center for Stem Cell Biology and Regenerative Medicine, University of Southern California, Los Angeles, CA 90033 USA; 2 Department of Biochemistry and Molecular Medicine, Keck School of Medicine, University of Southern California, Los Angeles, CA 90033 USA; 3 Children’s Hospital Los Angeles, Los Angeles, CA 90027 USA; 4 USC Caruso Department of Otolaryngology - Head and Neck Surgery, Keck School of Medicine, University of Southern California, Los Angeles, CA 90033 USA; 5 Department of Radiology, Keck School of Medicine, University of Southern California, Los Angeles, CA 90033 USA; 6 Center for Craniofacial Molecular Biology, Ostrow School of Dentistry, University of Southern California, Los Angeles, CA 90033 USA; 7 Fulgent Diagnostics, Temple City, CA 91780 USA

## Abstract

Whereas Jagged1-Notch2 signaling is known to pattern the sensorineural components of the inner ear, its role in middle ear development has been less clear. We previously reported a role for Jagged-Notch signaling in shaping skeletal elements derived from the first two pharyngeal arches of zebrafish. Here we show a conserved requirement for Jagged1-Notch2 signaling in patterning the stapes and incus middle ear bones derived from the equivalent pharyngeal arches of mammals. Mice lacking *Jagged1* or *Notch2* in neural crest-derived cells (NCCs) of the pharyngeal arches display a malformed stapes. Heterozygous *Jagged1* knockout mice, a model for Alagille Syndrome (AGS), also display stapes and incus defects. We find that Jagged1-Notch2 signaling functions early to pattern the stapes cartilage template, with stapes malformations correlating with hearing loss across all frequencies. We observe similar stapes defects and hearing loss in one patient with heterozygous *JAGGED1* loss, and a diversity of conductive and sensorineural hearing loss in nearly half of AGS patients, many of which carry *JAGGED1* mutations. Our findings reveal deep conservation of Jagged1-Notch2 signaling in patterning the pharyngeal arches from fish to mouse to man, despite the very different functions of their skeletal derivatives in jaw support and sound transduction.

## Introduction

**Figure 1:**
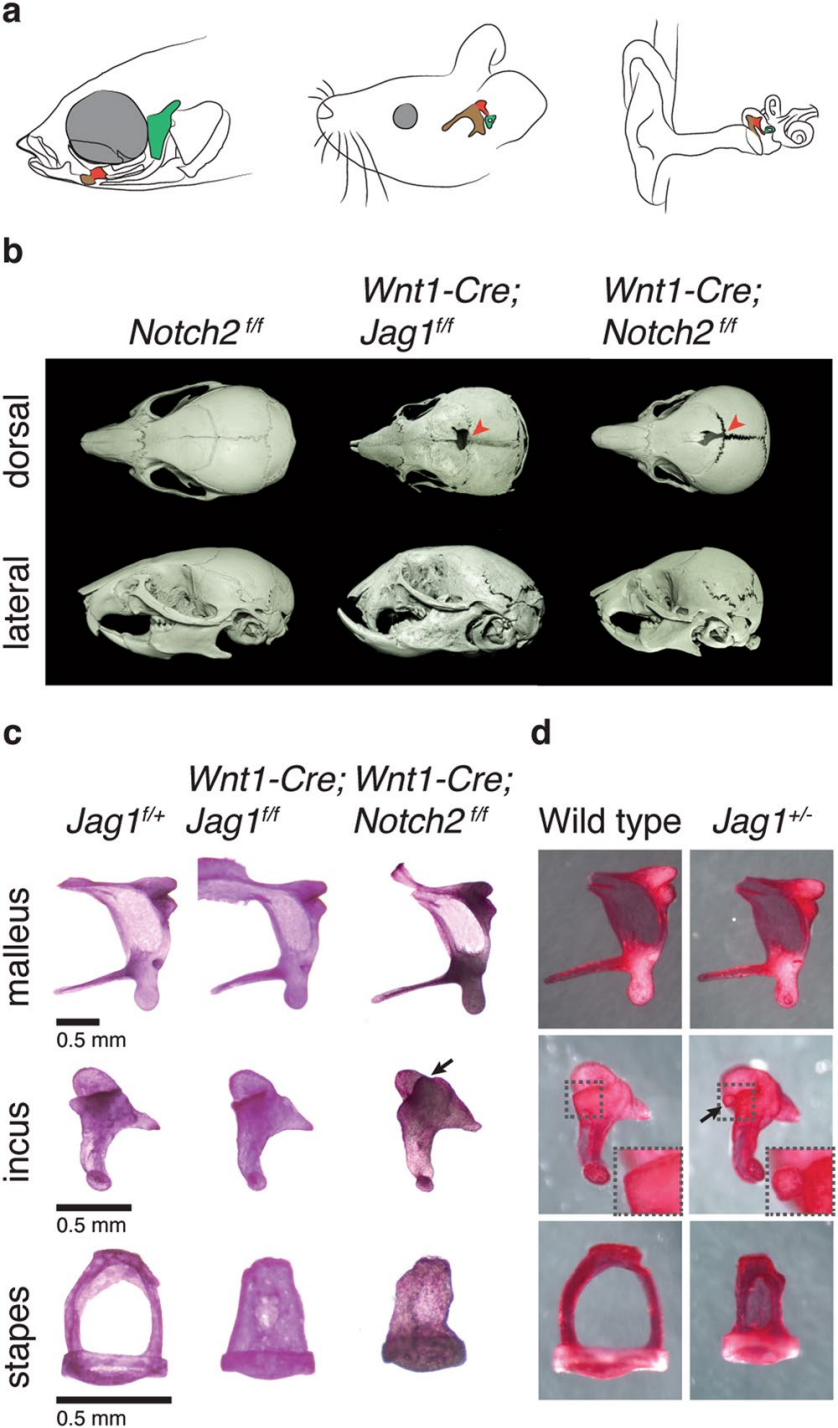
Craniofacial and middle ear defects in mice deficient for *Jag1* or *Notch2*. (**a**) Diagrams of the heads of zebrafish, mouse, and human (left to right) show homology between the fish jaw skeleton and the mammalian middle ear ossicles. The fish hyomandibula is homologous to the mammalian stapes (green); the fish palatoquadrate is homologous in part to the mammalian incus (red); and the proximal portion of the fish Meckel’s cartilage is homologous to the mammalian malleus (brown). (**b**) Micro-CT scans of mouse skulls at three weeks of age. Compared to control *Notch2* ^*f/f*^ mice, *Wnt1-Cre*; *Jag1* ^*f/f*^ and *Wnt1-Cre*; *Notch2* ^*f/f*^ mice exhibit a persistent foramen (arrowheads in dorsal view) and midfacial hyperplasia resulting in an abnormally shaped skull and malocclusion (lateral view). (**c**,**d**) Dissected middle ear ossicles of three-week-old mice stained with Alizarin Red S. Compared to *Jag1* ^*f/*+^ controls, *Wnt1-Cre*; *Jag1* ^*f/f*^ and *Wnt1-Cre*; *Notch2* ^*f/f*^ mice display a fully penetrant columellar stapes phenotype. *Wnt1-Cre*; *Notch2* ^*f/f*^ mice also rarely display an ectopic process from the anterior medial edge of the incus (arrow). Compared to wild-type siblings, some *Jag1* ^+/−^ mice display a columellar stapes and a small ectopic process from the posterior medial edge of the incus body (arrow and inset).

Despite their critical importance in sound transduction, we still know relatively little about the developmental patterning of the diminutive ossicles of the mammalian middle ear. The malleus and incus bones are derived from NCCs that populate the first (i.e. mandibular) pharyngeal arch, and the stapes bone is derived from the second (i.e. hyoid) arch^1^. In a fascinating evolutionary transition, these tiny middle ear bones are thought to have arisen by modification of the ancestral fish jaw-support skeleton, with the malleus, incus, and stapes being homologous to portions of the Meckel’s, palatoquadrate, and hyomandibular elements of fish, respectively^2^ (Fig. 1a). In a genetic screen in zebrafish, we previously identified a loss-of-function mutation in the *JAG1* homolog, *jag1b*, which resulted in specific malformations of the palatoquadrate and hyomandibular cartilages^3^. Subsequently, we found that Jag1b works through the Notch2 and Notch3 receptors to regulate bone and cartilage differentiation in the dorsal portions of the mandibular and hyoid arches, regions from which the incus and stapes bones arise in mammals^4^. We therefore asked in this study whether loss of Jagged-Notch signaling might similarly disrupt development of the stapes and incus bones.

In humans, heterozygous loss-of-function mutations in *JAGGED1* (*JAG1*) have been found in 94% of AGS patients^5–7^, with a small proportion of AGS patients harboring heterozygous loss-of-function mutations in *NOTCH2* ^8^, which encodes a receptor for JAG1. Clinical diagnosis of AGS is based on reduced numbers of intrahepatic bile ducts in the liver, accompanied by cholestasis, a characteristic facial appearance, and defects in the heart, eyes, and skeleton^9^. Although not part of the clinical diagnosis for AGS, there are some reports of hearing loss in patients with AGS and/or mutations in *JAG1*. Work in mouse and chick has uncovered roles for Jagged-Notch signaling in the patterning of the prosensory domain, which gives rise to the hair and support cells of the cochlea^10–12^. These findings would appear to suggest that hearing loss in AGS is primarily due to sensorineural defects. However, there are also a few isolated reports of conductive hearing loss in AGS, which indicates potential structural defects of the middle ear. In one large AGS kindred, mild conductive hearing loss was noted^13^. Another kindred, with missense mutations in *JAG1* yet only the cardiac defects typically associated with AGS, displayed mixed hearing loss (i.e. combined conductive and sensorineural components)^14^. In a separate post-mortem analysis of the temporal bone, two AGS individuals were described as having a “bulky” stapes, with one also displaying a “bulky” incus, yet the precise morphological changes of these middle ear bones were unclear^15^. These studies raise the possibility that defects in not only the neural components of the inner ear but also the structural components of the middle ear might contribute to hearing loss in AGS patients.

## Results

### Loss of *Jag1* or *Notch2* in NCCs results in craniofacial and middle ear defects

Homozygous deletion of *Jag1* or *Notch2* results in early embryonic lethality in mice^16, 17^. We had previously reported that conditional deletion of *Jag1* in NCCs (using *Wnt1*-*Cre*) resulted in a persistent foramen in the frontal bone^18^, and an independent group reported midfacial hypoplasia in these *Wnt1*-*Cre*; *Jag1* ^*f/f*^ conditional knockout (*Jag1*-CKO) mice, reminiscent of the facial characteristics of AGS^19, 20^. Micro-computed tomography (uCT) scans confirmed these previously reported phenotypes in *Jag1*-CKO mice, and revealed a similar persistent foramen and midfacial hypoplasia in *Wnt1*-*Cre*; *Notch2* ^*f/f*^ (*Notch2*-CKO) mice (Fig. 1b). We therefore analyzed the effects of removing both copies of *Jag1* or *Notch2* in NCCs on middle ear bone development. At postnatal day 21 (P21), Alizarin Red staining of bone in *Jag1* ^*f/f*^ controls shows that the stapes consists of two cruces with a prominent foramen. In 100% of *Jag1*-CKO (n = 8/8) and *Notch2*-CKO (n = 8/8) mice, the stapes was narrower than in controls and had a reduced or absent foramen (Fig. 1c and Table 1). Whereas the stapes of *Jag1*-CKO mice were consistently reduced in size, there was some variability in the amount of ectopic mineralization in the reduced foramen (Fig. S1). This single crus phenotype of the stapes has previously been referred to as “columellar” or “monopode”^21–24^. In contrast, the malleus and incus bones of *Jag1*-CKO and *Notch2*-CKO mice were less affected, with just 12.5% (n = 1/8) of *Notch2*-CKO mice and no *Jag1*-CKO mice having an ectopic process extending from the anterior medial edge of the incus body.

**Table 1:** Summary of incus and stapes defects in mice deficient for *Jag1*, *Notch2*, and *Twist1*.

Genotype	N	Incus		Stapes	N	Retrotympanic process
		ectopic posterior medial process	ectopic anterior medial process	small or no lumen		broken
Wild type	18	3 (16.7%)	0 (0%)	0 (0%)	23	1 (4%)
*Jag1* ^*f/*+^	9	0 (0%)	0 (0%)	0 (0%)	N.D.	—
*Wnt1-Cre*; *Jag1* ^*f/*+^	9	0 (0%)	0 (0%)	0 (0%)	N.D.	—
*Wnt1-Cre*; *Jag1* ^*f/f*^	8	1 (12.5%)	0 (0%)	8 (100%)^d^	N.D.	—
*Notch2* ^*f/*+^	5	0 (0%)	0 (0%)	0 (0%)	N.D.	—
*Wnt1-Cre*; *Notch2* ^*f/*+^	7	0 (0%)	0 (0%)	0 (0%)	N.D.	—
*Wnt1-Cre*; *Notch2* ^*f/f*^	8	0 (0%)	1 (12.5%)	8 (100%)^e^	N.D.	—
*Jag1* ^+/−^	20	15 (75%)^a^	0 (0%)	5 (25%)^f^	15	0 (0%)
*Twist1* ^+/−^	16	0 (0%)	6 (38%)^b^	0 (0%)	18	2 (11%)
*Jag1* ^+/−^; *Twist1* ^+/−^	13	0 (0%)	9 (69%)^c^	1 (8%)	22	15 (68%)^g^

For statistical analysis, we performed a multi-group comparison using a Fisher Exact Test or Chi-Square Test, followed by post-hoc pair-wise comparisons. N.D. =not determined.

^a^p < 0.005 vs wild type, *Twist1* ^+/−^, and *Jag1* ^+/−^; *Twist1* ^+/−^.

^b^p < 0.01 vs wild type and *Jag1* ^+/−^.

^c^p < 0.025 vs *Twist1* ^+/−^.

^d^p < 0.001 vs *Jag1* ^f/+^ and *Wnt1*-*Cre*; *Jag1* ^f/+^.

^e^p < 0.001 vs *Notch2* ^f/+^ and *Wnt1*-*Cre*; *Notch2* ^f/+^.

^f^p < 0.05 vs wild type.

^g^p < 0.001 vs wild type, *Jag1* ^+/−^ and *Twist1* ^+/−^.

As heterozygous loss-of-function mutations in human *JAG1* result in AGS, we also examined the effects of removing just one copy of *Jag1* throughout the whole mouse (Fig. 1d and Table 1). In *Jag1* heterozygotes, we observed a similar columellar stapes in 25% of animals (n = 5/20), and an ectopic process extending from the posterior medial edge of the incus body in 75% of animals (n = 15/20). As with *Jag1*-CKO mice, the malleus was unaffected in *Jag1* heterozygotes. These findings indicate that development of the stapes and incus is especially sensitive to reduced dosage of Jag1-Notch2 signaling in mice.

### *Jag1* is necessary for early patterning of the stapes cartilage but not formation of the stapedial artery

**Figure 2:**
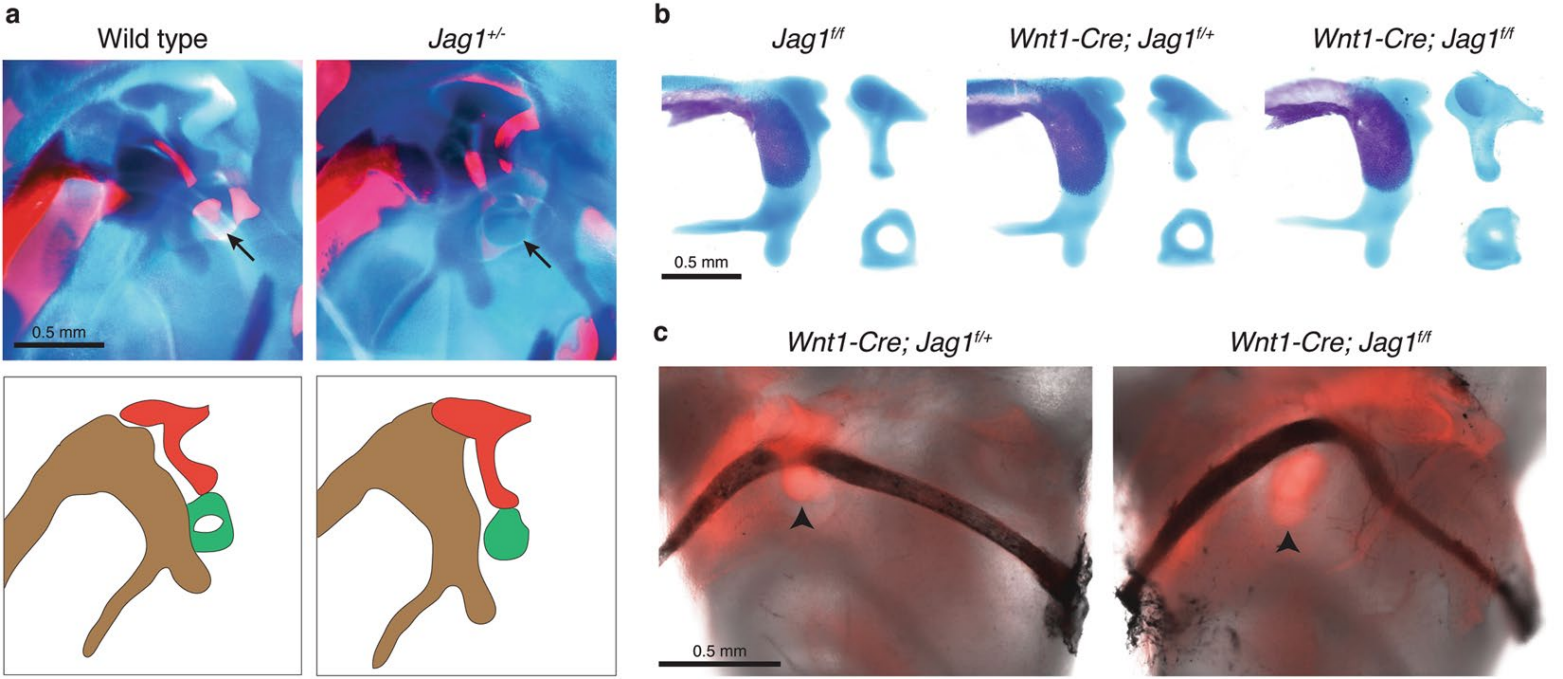
Mispatterning of middle ear cartilages and formation of the stapedial artery in *Jag1*-deficient mice. (**a**,**b**) Newborn mice were stained with Alcian Blue for cartilage and Alizarin Red S for bone. Close-up views show the developing middle ear, which is diagrammed below for wild-type and *Jag1* heterozygous mice (malleus, brown; incus, red; stapes, green). Dissected middle ear cartilages are shown for conditional mutants. Arrows point to the stapes cartilage, which is reduced in size in both heterozygous and conditional *Jag1* mutant mice. (**c**) The stapes of *Wnt1-Cre*; *Jag1* ^*f/*+^; Rosa26-Tomato and *Wnt1-Cre*; *Jag1* ^*f/f*^; Rosa26-Tomato mice fluoresce red and the stapedial arteries appear black from India ink injection. The artery is still present in *Jag1*-CKO mice, where it deviates around the misshapen stapes cartilage. Arrowheads point to the stapes.

The lack of a foramen in the mutant stapes could be due to earlier mispatterning of the cartilage template, ectopic mineralization, or loss of the stapedial artery that runs through the normal foramen. We therefore examined the middle ear cartilages of newborn mice, before they are ossified, using Alizarin Red and Alcian Blue to stain for bone and cartilage (Fig. 2a,b and Table 1). In wild-type, *Jag1* ^*f/f*^, and *Wnt1-Cre*; *Jag1* ^*f/*+^ controls, the stapes cartilage had a prominent foramen. In contrast, *Jag1* ^+/−^ and *Wnt1*-*Cre*; *Jag1* ^*f/f*^ mice had a smaller stapes cartilage with a reduced or absent foramen, consistent with the later columellar phenotype of the ossified stapes bone. We next examined the stapedial artery, which normally passes through the stapes and serves as a bridge connecting the external and internal carotid arteries. To visualize this artery in conjunction with the stapes cartilage, we bred the conditional *Jag1* mutants onto a Rosa26-Tomato reporter background and injected India ink into the artery after dissecting out the intact middle and inner ear from newborn mice. As in controls, we still observed a prominent stapedial artery in *Jag1*-CKO mice (n = 3/3), which curved around the misshapen stapes cartilage (Fig. 2c). These findings indicate that the stapes defects in *Jag1*-deficient mice are likely due to an early mispatterning of the cartilage template rather than loss of the stapedial artery or ectopic mineralization.

### *Jag1* and *Twist1* interact to pattern the incus and retrotympanic process

**Figure 3:**
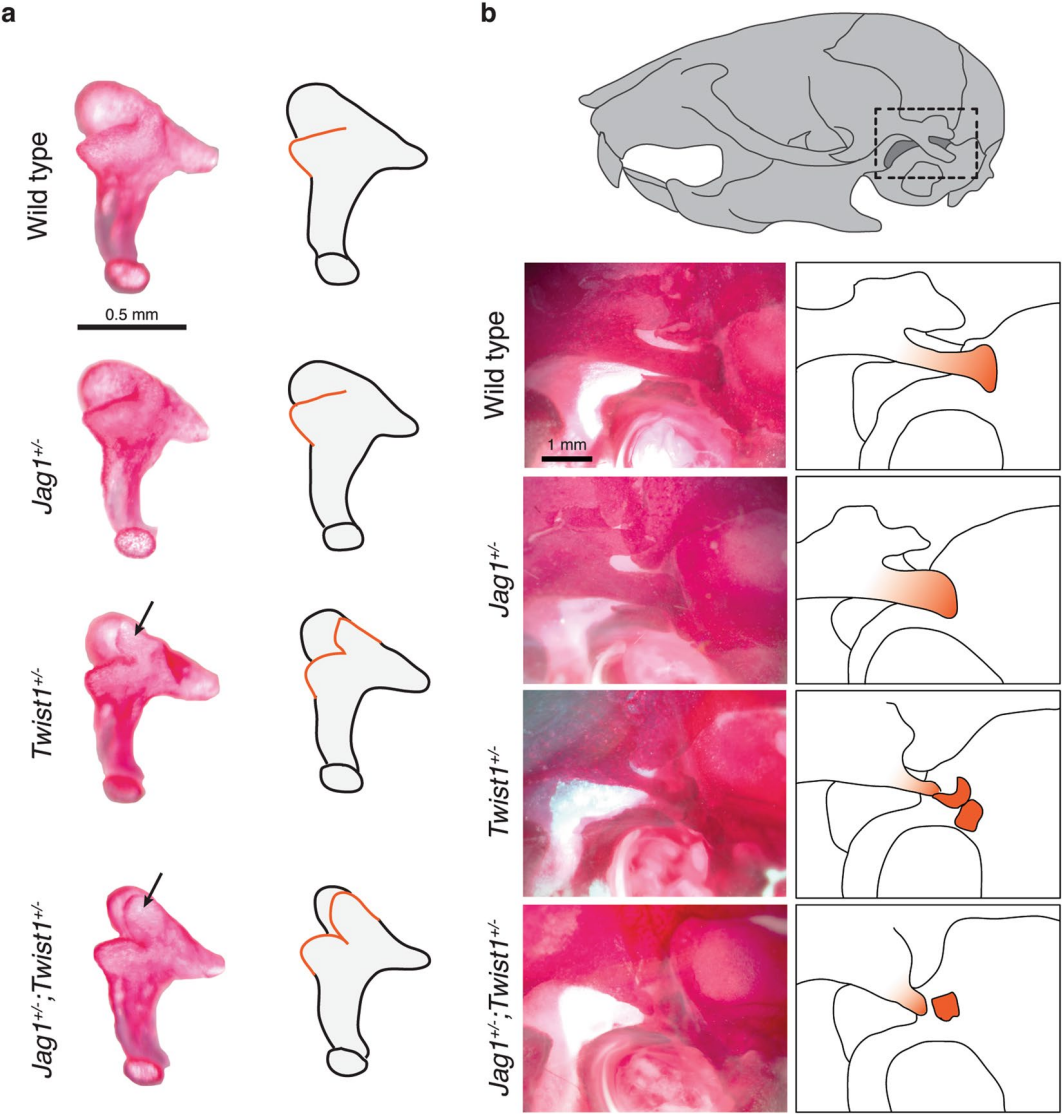
Incus and retrotympanic defects in *Jag1*; *Twist1* compound mutants. (**a**) Dissected incus bones of three-week-old mice stained with Alizarin Red S. Wild types and this *Jag1* ^+/−^ example display a normal incus. In contrast, *Twist1* ^+/−^ and *Jag1* ^+/−^; *Twist1* ^+/−^ mice have an extra process (black arrows) extending from the anterior medial edge of the incus body. Accompanying diagrams illustrate the ectopic processes with orange lines. (**b**) Views of the temporal bone in three-week-old mice stained with Alizarin Red S. The dashed box in the illustration shows the approximate region being imaged. Compared to wild-type and *Jag1* ^+/−^ mice, *Twist1* ^+/−^ and *Jag1* ^+/−^; *Twist1* ^+/−^ mice display reduction and fragmentation of the retrotympanic process (shown in orange in the adjacent diagrams).

Because we had previously uncovered a genetic interaction between *Jag1* and *Twist1* in coronal suture development^18^, we next examined whether the variable penetrance of middle ear defects in *Jag1* heterozygotes might be due in part to genetic interactions with *Twist1*. In humans, heterozygous loss of *TWIST1* results in Saethre-Chotzen syndrome^25^, a variable feature of which is conductive or mixed hearing loss^26, 27^. In *Twist1* ^+/−^ mice, we observed that the stapes and malleus bones were normal, with loss of one *Twist1* allele failing to enhance the stapes defects of *Jag1* ^+/−^ mice (Table 1). In contrast, 38% (n = 6/16) of *Twist1* ^+/−^ mice developed a prominent ectopic process from the anterior medial edge of the incus body, and in *Jag1* ^+/−^; *Twist1* ^+/−^ mice the penetrance of this phenotype increased to 69% (n = 9/13) (Table 1 and Fig. 3a). The retrotympanic process, a posterior extension of the squamosal bone that lies just above the incus, was also reduced in size and fragmented in *Twist1* ^+/−^ but not *Jag1* ^+/−^ single mutants. The penetrance of this phenotype increased from 11% (n = 2/18) to 68% (n = 13/22) in compound heterozygotes (Table 1 and Fig. 3b). These findings reveal a selective interaction between *Jag1* and *Twist1* in patterning the mandibular arch from which the incus and retrotympanic process derive, and not the hyoid arch from which the stapes derives. As both Twist1 and Jagged-Notch signaling inhibit skeletal differentiation in the head^18, 28–30^, inappropriate skeletal differentiation might underlie both the suture and middle ear bone phenotypes in animals deficient in these factors. Further analysis will be required to determine whether Twist1 and Jagged-Notch signaling function in a linear pathway or in parallel for mandibular arch patterning.

### *Jag1* is required in NCCs for normal hearing in mice

**Figure 4:**
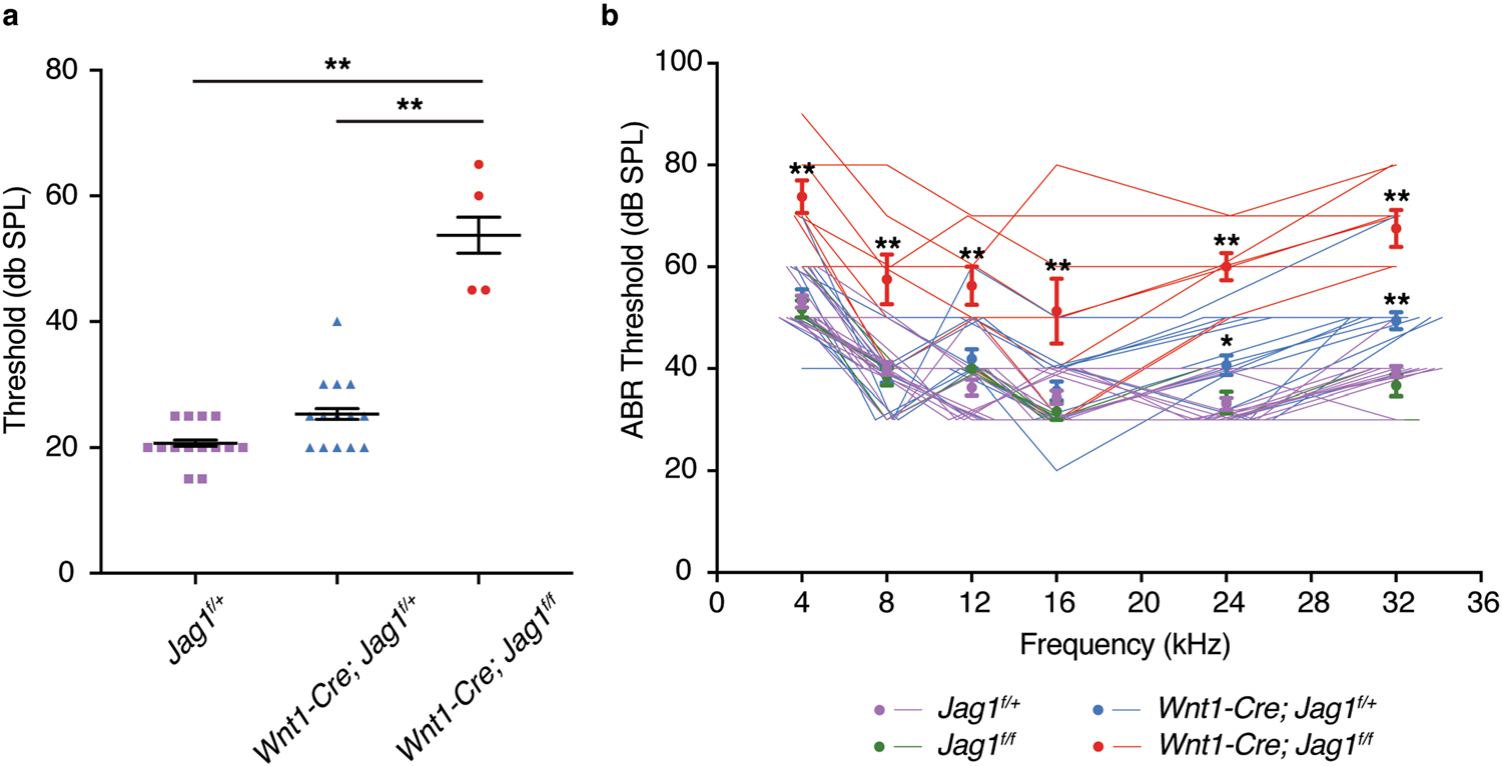
Hearing loss in mice lacking *Jag1* in NCCs. (**a**) A click stimulus test was performed in P18 mice, a stage at which wild-type mice show normal hearing. Compared to *Jag1* ^*f/*+^ (*n* = 7) and *Wnt1-Cre*; *Jag1* ^*f/*+^ (*n* = 7) mice, the threshold at which *Wnt1-Cre*; *Jag1* ^*f/f*^ mice (*n* = 2) could hear was significantly higher. Each point represents one individually tested ear. **p < 0.01; differences were measured by one-way ANOVA with post-hoc Tukey-Kramer HSD test. Error bars represent standard error of the mean. (**b**) Auditory brainstem responses were recorded at a range of frequencies in P18 mice. Compared to *Jag1* ^*f/*+^ (*n* = 8), *Jag1* ^*f/f*^ (*n* = 3), and *Wnt1-Cre*; *Jag1* ^*f/*+^ (*n* = 8) mice, *Wnt1-Cre*; *Jag1* ^*f/f*^ mice (*n* = 4) showed significantly higher thresholds across all frequencies as determined by a one-way ANOVA and subsequent post-hoc Tukey-Kramer HSD test. Hearing thresholds of *Wnt1-Cre*; *Jag1* ^*f/*+^ mice were similar to *Jag1* ^*f/*+^ and *Jag1* ^*f/f*^ control mice at lower frequencies but significantly different from *Jag1* ^*f/*+^ controls at 24 and 32 kHz. Circles represent averages, and lines represent individually tested ears. *p < 0.05, **p < 0.01; error bars represent standard error of the mean.

The stapes and incus bones are essential for normal hearing^31, 32^. To test whether the ossicular defects of *Jag1*-CKO mice result in hearing loss, we measured auditory brainstem response (ABR) at P18. In a click stimulus test, in which a large range of frequencies is presented simultaneously, we observed an approximately 30 decibel shift in the sound pressure level (dB SPL) in *Jag1*-CKO mice compared to *Wnt1-Cre*; *Jag1* ^*f/*+^ or *Jag1* ^*f/*+^ controls (Fig. 4a). Hearing level thresholds were then measured at the specific frequencies of 4, 8, 12, 16, 24, and 32 kilohertz (kHz). We found a roughly 22 db SPL threshold shift in *Jag1*-CKO mice across all frequencies, significantly higher than *Wnt1-Cre*; *Jag1* ^*f/*+^, *Jag1* ^*f/*+^, and *Jag1* ^*f/f*^ mice (Fig. 4b). We also found a small but statistically significant high-frequency threshold shift in mice lacking just one copy of *Jag1* in NCCs, compared to *Jag1* ^*f/*+^ controls; however this shift appeared to be largely attributable to one animal and was no longer apparent when mice were retested at five weeks of age (Fig. S2). As we deleted *Jag1* solely in NCCs, these results are consistent with hearing loss being due to structural defects of the stapes and/or other components of the middle ear. It remains unclear whether the variability in hearing acuity of individual *Jag1*-CKO animals could be explained by the small differences in stapes morphology observed (Fig. S1). As neural crest-derived cells also make a small contribution to the inner ear, defects in these structures might also contribute to the degree of hearing loss. However, the semicircular canals of the inner ear, which are dysmorphic in Alagille Syndrome^14^ and conventional *Jag1* heterozygous mice^11, 33, 34^, were unaffected in *Jag1*-CKO mice (Fig. S3), arguing against canal defects being the cause of hearing loss.

### Conductive hearing loss and anomalies in middle ear bones in patients heterozygous for *JAG1* loss-of-function mutations

**Figure 5:**
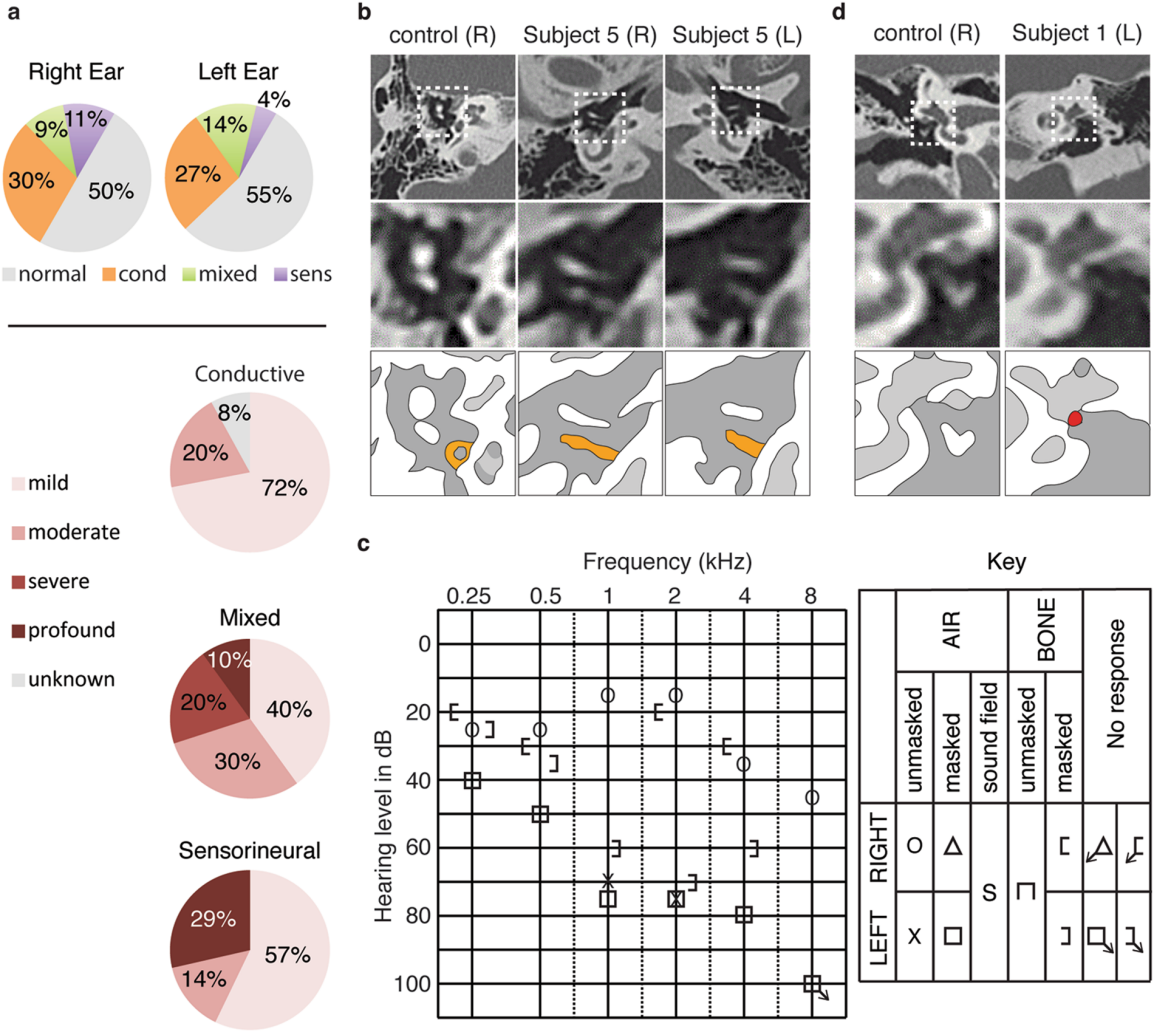
Hearing loss and middle ear defects in patients heterozygous for *JAG1* mutations and/or diagnosed with AGS. (**a**) The ratio of types of hearing loss for the right and left ears were calculated based on the 44 subjects tested. The degrees of hearing loss encompassed in each type of hearing loss were also analyzed. Table S1 lists the degrees of hearing loss as a range indicating the loss at its best frequency and worst frequency. The categorical analyses illustrated by the pie charts have taken into account only the loss at the worst frequency. (**b**) CT scans of the temporal bone in the axial plane from a control 69-year-old male without AGS and subject 5 who is heterozygous for a *JAG1* loss-of-function mutation. Magnified areas of the dashed box regions and accompanying diagrams are shown below. Compared to the right stapes (orange) from the control subject, the right and left stapes of subject 5 appear as a single rod (i.e. columellar). Adjacent sections showed relatively normal articulation of the stapes with the incus. For better comparison, the orientation of the left stapes is flipped horizontally in the magnified view. (**c**) Audiogram of subject 5 (see Table 2) indicates mild to profound mixed hearing loss in the left ear and normal to mild sensorineural hearing loss with a potential high-frequency conductive component in the right ear. (**d**) Compared to a control 69-year-old male without AGS, CT scans of the temporal bone in the coronal plane show inappropriate ossification (red) of the oval window in the left ear of subject 1. The control right ear is flipped horizontally in the magnified area and accompanying diagram.

Given the stapes defects in heterozygous *Jag1* mutant mice, we investigated whether heterozygous loss of *JAG1* might also affect middle ear development and hearing in humans. Conductive and mixed hearing loss have been described in a few kindreds with mutations or deletions in *JAG1* ^13–15^, yet the prevalence of such hearing loss in AGS was unclear^35^. We therefore attended the Alagille Alliance meetings in 2011 and 2014 and conducted hearing tests on participants with AGS clinical diagnosis and/or known heterozygous mutations in *JAG1*. Of the 44 subjects tested, 16 have known mutations in *JAG1* and the remaining 28 have not yet been determined (Table S1). The most common finding was conductive hearing loss (27% of left ears, 30% of right ears), followed by mixed hearing loss (14% of left ears, 9% of right ears) and then sensorineural hearing loss (4% of left ears, 11% of right ears) (Fig. 5a and Table S1). We found conductive hearing loss to be primarily mild or moderate, compared to sensorineural and mixed hearing loss, which could be in the severe to profound range (Fig. 5a).

For five participants – one with conductive, two with mixed, one with sensorineural, and one with no hearing loss – and a non-AGS control, we obtained computed tomography (CT) scans of the temporal bone to visualize middle ear structures (summarized in Table 2). CT scans have been shown to be fairly accurate in diagnosing stapes defects^36^. All five subjects showed abnormalities in the posterior semicircular canals. Three showed defects in the superior semicircular canals, consistent with previous reports of hypoplasia or absence of semicircular canals in AGS^14, 37^. In a 49-year-old male with severe mixed hearing loss in the left ear and high-frequency hearing loss in the right ear, we observed bilateral malformations of the stapes such that it appeared columellar (i.e. lacking distinct anterior and posterior cruces) (Fig. 5b,c), nearly identical to what we observed in *Jag1*-deficient mice. This subject has a mutation in exon 19 (c.2345–2A > G) of *JAG1* that alters the splicing consensus sequence and leads to heterozygous *JAG1* loss-of-function. He also had normal compliance of the tympanic membrane in both ears. These findings are consistent with partial defects in the ossicular chain resulting in high-frequency hearing loss^38^. We also observed an ectopic process extending from the left incus towards the posterior wall of the tympanic cavity in a 9-year-old subject with sensorineural hearing loss in the right ear, and irregular orientation of the petrous part of the temporal bone, which was sloping upwards in a lateral to medial manner, in an 8-year-old subject with mixed hearing loss in both ears (Table 2). In a 7-year-old subject with mild low-frequency conductive hearing loss in the left ear, middle ear bones were normal yet the aperture of the cochlear nerve was reduced. A 16-year-old subject with normal hearing, who had no defects in the middle ear bones, also displayed abnormal calcification of the left oval window (Fig. 5d). This lesion is suggestive of otosclerosis, in which calcified bone fixes the stapes to the oval window and in some cases limits the ability to transmit sound. In summary, while conductive hearing loss was associated with stapes defects in one subject, our findings show a diversity of structural defects of the middle ear in AGS patients that are variably associated with hearing loss.

**Table 2:** Hearing tests and CT data of selected subjects from the 2011 and 2014 Alagille Alliance meetings.

Subject	Age	Mutation	Ear	Audiologic Data			CT Data
				Type of Hearing Loss	Degree of Hearing Loss	Tympanogram	Affected Structures
1	16	*JAG1*	R	—	—	normal	PSCC, CoA
			L	—	—	normal	OW, PSCC, CoA
2^a^	9	*JAG1*	R	sens	mild	normal	SSCC, PSCC
			L	—	—	normal	incus
3	8	*JAG1*	R	mixed	mild	negative ME pressure/normal compliance	OW, RW, SSCC, PSCC
			L	mixed	mild-severe	negative ME pressure/normal compliance	OW, RW, SSCC, PSCC
4	7	N.D.	R	—	—	normal	PSCC, CoA
			L	cond	mild	normal	PSCC
5^b^	49	*JAG1*	R	sens	normal-mild	normal	stapes, SSCC, PSCC, CoA
			L	mixed	mild-profound	normal	stapes, SSCC, PSCC, CoA

Cond = conductive hearing loss; CoA = cochlear aperature; CT = computed tomography; L = left; ME = middle ear; N.D. =not determined OW = oval window; PSCC = posterior semicircular canal; R = right; RW = round window; sens = sensorineural hearing loss; SSCC = superior semicircular canal;- = within normal limits.

^a^Previous test had showed conductive hearing loss in right ear.

^b^This subject has mutations in *JAG1* but had not previously been diagnosed with AGS.

## Discussion

Our findings indicate a conserved role for Jag1-Notch2 signaling in NCCs for the patterning of the stapes and incus bones of the mammalian middle ear. The fully penetrant stapes defects seen upon NCC-specific deletion of *Jag1* or *Notch2* are consistent with findings in zebrafish that Jag1b functions in NCCs to pattern the homologous hyomandibular cartilage^3^. Whereas hyomandibular defects in zebrafish lacking *jag1b*, or *notch2* and *notch3*, correlate with ectopic expression of the cartilage condensation marker *barx1* in the hyoid arch^4^, we observed no differences in *Barx1* expression in the hyoid arches of *Jag1*-CKO mice at E10.5 and E11.5 (Fig. S4). However, it remains possible that subtle differences in *Barx1* expression escaped our detection, especially given the small number of arch NCCs that contribute to the diminutive stapes bone. On the other hand, it seems less likely that stapes defects are a secondary consequence of stapedial artery defects. *Jag1* has been shown to be required in endothelial cells, which are not of neural crest origin, for vascular development^39^. Further, we show that NCC-specific loss of the Notch2 receptor, which is expected to act cell-autonomously in endothelial cells and not NCCs for artery development, causes a similar stapes defect to NCC-specific *Jag1* loss. Whereas the stapedial artery is later associated with pericytes, which are of neural crest origin, pericytes are recruited only after artery formation and thus are unlikely to affect the initial routing of the stapedial artery^40^. Further, the foramen of the stapes homolog (hyomandibula) in zebrafish *jag1b* mutants is similarly lost, despite this foramen being associated with the facial nerve instead of an artery^3^. Thus, rerouting of the stapedial artery appears to be a secondary consequence of the reduced stapes anlagen and not vice versa. Nonetheless, additional studies are clearly needed to elucidate the developmental basis of stapes defects in *Jag1*-deficient mice.

*Wnt1-Cre*; *RBP-J* ^*f/f*^ mice with global loss of Notch signaling in NCCs display similar defects in the frontal bone as we observe upon NCC-specific deletion of *Jag1* or *Notch2* ^41^. Although middle ear bones were not examined in *Wnt1-Cre*; *RBP-J* ^*f/f*^ mice, the similarity of stapes defects in *Jag1*-CKO and *Notch2*-CKO mice suggest that JAG1 and NOTCH2 are the likely major ligand and receptor for both calvarial and middle ear bone development. Of note, global but not NCC-specific heterozygous loss of *Jag1* results in partially penetrant stapes defects, as well as incus defects, suggesting additional requirements for *Jag1* in non-NCC tissues for ossicle patterning. One candidate tissue is the first endodermal pouch, which displays strong *Jag1* expression in both mice^42^ and zebrafish^3^ and develops in close association with the stapes and incus anlagen. *Jag2* and *Notch3* may also partially compensate for *Jag1* and *Notch2* in other aspects of NCC skeletal differentiation. *Jag2* is required for development of the NCC-derived palate in mice^43^, and *notch3* reduction enhances craniofacial defects in *notch2* mutant zebrafish^4^. However, deletion of *Notch1* in neural-crest-derived cells does not cause midfacial hypoplasia characteristic of Alagille Syndrome^20^, and a previous study did not detect expression of either *Notch1* ortholog in the developing zebrafish face^3^. Interestingly, the liver and heart defects of AGS have not been observed in the analogous *Jag1* heterozygous mice^17, 44^, yet we did observe similar stapes defects in both mice and humans lacking one copy of *JAG1*. These results indicate that organs have independent dosage sensitivities to *JAG1* across species.

While we found a correlation between stapes defects and hearing loss in one individual heterozygous for a *JAG1* mutation, the presence of conductive or mixed hearing loss but apparently normal middle ear bones in other AGS patients suggests that middle ear bone defects alone cannot account for conductive hearing loss in this syndrome. NCC-specific *Jag1* mutant mice have midfacial hypoplasia, and the development and function of the Eustachian tube is closely associated with the craniofacial skeleton^45, 46^. Thus, other factors such as Eustachian tube dysfunction may contribute to hearing loss in subjects with apparently normal ossicles. Similarly in NCC-specific *Jag1* mutant mice, we cannot rule out the possibility that defects in other NCC-derived ear structures besides the stapes contribute to hearing loss, especially as the ABR test we performed does not distinguish between conductive and sensorineural components. For example, NCC derivatives contribute to the early cochleovestibular ganglion and utricle, as well as a few rare cells in the semicircular canals and stria vascularis^47^. While the cochleovestibular ganglion and stria vascularis are involved in auditory functions, the utricle and semicircular canals are responsible for balance. Indeed, studies on three mouse mutants – *Slalom* ^33^, *Headturner* ^48^, and *Ozzy* ^34^ – have described specific point mutations in *Jag1* resulting in malformations of the semicircular canals, and we and others observe frequent losses and anomalies of the superior and posterior semicircular canals in AGS patients^14, 15, 37^. Further, the role of Jagged-Notch signaling in the development of the vestibular system also appears to be conserved in zebrafish, as *jag1b* mutants were independently isolated based on semicircular canal defects^49, 50^. However, we found that NCC-specific loss of *Jag1* in mice causes hearing loss without affecting canal morphology, consistent with defects in the stapes and/or other middle and inner ear structures being responsible for hearing defects in these mice. Nonetheless, superior semicircular canal dehiscence can lead to autophony and conductive hearing loss, and dehiscence of the canal can cause disruption of the normal endolymph flow, resulting in a lower bone conduction and higher air conduction threshold^51^. Although there are currently no reports linking *Jag1* and AGS to canal dehiscence, we cannot rule out a possible canal dehiscence component in AGS-associated hearing loss. In addition, *Jag1* has a well-known requirement in patterning the ectodermal sensory placode from which the hair and support cells of the inner ear derive^10–12^, consistent with the finding of sensorineural hearing loss in several AGS patients. These observations indicate a complex etiology of hearing loss in AGS, likely affecting multiple structures in both the middle and inner ear, particularly for those patients presenting with mixed hearing loss. However, for those patients with isolated middle ear bone defects, surgical correction might be an option to improve hearing^52^.

To date, mutations in only a few genes, such as *NOGGIN* ^53^ and *ANK* ^54^, have been linked to congenital conductive hearing loss in humans. A contribution of *JAG1* mutations to congenital conductive hearing loss thus expands our knowledge of human middle ear development. However, as AGS is relatively rare, we were only able to examine the middle ears of a small number of patients. Thus, the extent to which stapes and/or incus defects occur in this syndrome and contribute to hearing loss remains to be determined. While the similarity in stapes defects between *Jag1* mutant mice and an individual with heterozygous loss of *JAG1* strongly supports the view that human stapes malformations are due to *JAG1* deficiency, we cannot rule out that second site mutations act synergistically with *JAG1* mutations to cause middle ear bone defects. For example, conditional deletion studies in mice have revealed requirements for Tbx1 in middle and inner ear structures^55^, and DiGeorge Syndrome, which is associated with heterozygous deletion of *TBX1*, encompasses an array of defects that overlap with Alagille Syndrome, including tetralogy of fallot and hearing loss. On the other hand, the patient with stapes defects did not display the multi-organ defects typically associated with AGS, despite having a child with the same *JAG1* mutation and typical AGS features. Together with a previous study showing mixed hearing loss in a kindred with a *JAG1* missense mutation but only a subset of AGS features^14^, and the presence of *JAG1* mutations presenting with only one or two AGS features^56^, our results suggest that reduced *JAG1* function can cause hearing loss largely independently of other AGS features. It will therefore be informative to examine the extent to which family members of AGS patients who exhibit hearing loss despite the lack of an AGS diagnosis, as well as unrelated patients with defects in the stapes and/or incus bones, carry *JAG1* or *NOTCH2* mutations.

## Materials and Methods

### Mouse mutants and genotyping

Animal experiments were approved by the University of Southern California IACUC committee, and all methods were performed in accordance with the relevant guidelines and regulations. Genotyping was performed as previously published for *Jag1* ^17^, *Twist1* ^57^, *Jag1*-flox^58^, *Notch2*-flox^59^, *Wnt1-Cre* ^60^, and Rosa26-Tomato^61^ mouse lines.

### Micro-computed tomography of mice

Imaging was performed using a MicroCT 50 (Scanco Medical AG, Switzerland), scanning at high resolution [2040 × 2040 in-plane image matrix; 0.18 degree rotational step (DRS)] and a field of view of 20.4 mm. Scans were conducted at an energy setting of 70 kVp, current intensity of 200 µA, and an integration time of 500 ms/projection. Two-dimensional slices taken at 10-micron increments were rendered into three-dimensional reconstructions using Exposure Render^62^.

### Skull preparation

The heads of three-week-old mice were skinned and cleared with 1% KOH for 1 to 2 days, stained with 2% Alizarin Red S in 1% KOH until mineralized bone is red, and stored in 100% glycerol. Middle ear ossicles were then dissected out for analysis. The heads of P0 newborn mice were skinned and double stained with Alcian Blue and Alizarin Red S for cartilage and bone as described^63^ with minor modifications. Washes in tap water pre and post bone staining were omitted; Alizarin Red S at 0.1% was used at 1:50 dilution in potassium hydroxide (KOH); decolorizing treatment after bone staining was performed with 1% KOH overnight; and the dehydration process was completed through sequentially increasing proportions of 100% glycerol:1% KOH (1:3, 1:1, 3:1, 1:0). Samples were imaged using a Leica S8 APO stereo microscope.

### Stapes and stapedial artery visualization

*Wnt1-Cre*; *Jag1* ^*f/*+^; Rosa26-Tomato^ki/+^ and *Jag1* ^*f/f*^; Rosa26-Tomato^ki/ki^ mice were bred to generate *Wnt1-Cre*; *Jag1* ^*f/f*^; Rosa26-Tomato mutants. The heads of P0 newborn mice were skinned, fixed overnight in 4% paraformaldehyde, and stored in phosphate buffered saline (PBS). Whole ear structures were carefully dissected out, and the stapedial artery was injected with India ink using glass capillary needles. The malleus, incus, and other tissues were removed after injections and before imaging to better reveal the stapes. Image z-stacks were taken using a Zeiss Axiozoom and processed using extended depth of field by Zeiss LSM software.

### Hearing tests in mice

ABR was performed through inserted earphones, using closed-field acoustics. The sound pressure level (SPL) of the stimuli ranged between 20 and 105 decibels (dB). In determining ABR thresholds, 300 responses with artifacts less than 30 microvolts were averaged. Presentation of stimuli and averaging of responses were both controlled by BioSig software. When ABR threshold was above maximum output range, it was classified as 105 dB. Hearing level thresholds were measured at specific frequencies of 4, 8, 12, 16, 24, and 32 kilohertz (kHz).

### Hearing tests and computed tomography (CT) scans on human subjects

Studies on human patients were approved by the Institutional Review Board at the University of Southern California Keck School of Medicine, and all methods were performed in accordance with the relevant guidelines and regulations. Written informed consent to obtain samples for genetics research was obtained from each subject and/or subject’s parent or guardian. Hearing tests were performed at the Alagille Alliance meetings of 2011 and 2014. Otoscopy was performed to check for occluding wax, fluid, or infection, as well as wellness of the canal and middle ear system. The testing used was tympanometry and behavioral audiometry. For tympanometry, a small probe tip is inserted into the ear canal to create a seal, and then air pressure is directed into the external auditory ear canal. The mobility of the eardrum is recorded to confirm the health of the middle ear system. For behavioral audiometry, thresholds of hearing are tested from 250 to 8000 Hz. Test procedures are based on the age of the test subject. If a subject is able to test conventionally by raising their hand only when a test frequency is heard, the standard 10 dB up and 5 dB down testing procedure is performed. Frequencies are tested in conventional order − 1000, 2000, 4000, 8000, 250, 500 Hz. If the difference between octaves is greater or equal to 20 dB, then inner octave threshold is measured. In cases where a subject is too young, unreliable or distracted to complete this testing procedure, conditioned play audiometry is completed. In this form of testing, the subject is trained to perform an action in response to sound, such as dropping a block in a bucket. Frequencies are not presented in any standard order to keep the subject interested. If the patient becomes unreliable, then thresholds are not recorded. In standard air and bone conduction tests, a hearing level of -10 to 25 dBHL (decibels hearing level) is classified as normal, 25 to 40 dBHL as mild, 40 to 70 dBHL as moderate, 70 to 90 dBHL as severe, and 90 dBHL and above as profound. Conductive hearing loss is indicated by decreased air but normal bone conduction, sensorineural hearing loss by decreased air and bone conduction thresholds, and mixed hearing loss by decreased air and bone conduction thresholds with the bone conduction threshold being 10 dB higher than for air. Temporal bone CT scans on subjects 1 and 5 were acquired at the University of Southern California Keck School of Medicine using standard clinical procedures. Temporal bone CT scans for subjects 2, 3, and 4 were kindly provided by the patients’ physicians.

### Data Availability

All data generated or analyzed during this study are either included in this published article or available from the corresponding author on request.

## Electronic supplementary material

Supplementary Information
