# Supplementary material for: Requirement for Jagged1-Notch2 signaling in patterning the bones of the mouse and human middle ear
Source: Sci Rep. 2017 May 31;7:2497. doi: 10.1038/s41598-017-02574-7 (PMC5451394; doi:10.1038/s41598-017-02574-7)
Supplement: Supplementary file 1 — Supplementary Information [file 41598_2017_2574_MOESM1_ESM.pdf]

Supplementary Information Related To “Requirement of Jagged1-Notch2 signaling in patterning the bones of the mouse and human middle ear”.

Camilla S. Teng, Hai-Yun Yen, Lindsey Barske, Bea Smith, Juan Llamas, Neil Segil, John Go, Pedro A. Sanchez-Lara, Robert E. Maxson, Jr., J. Gage Crump

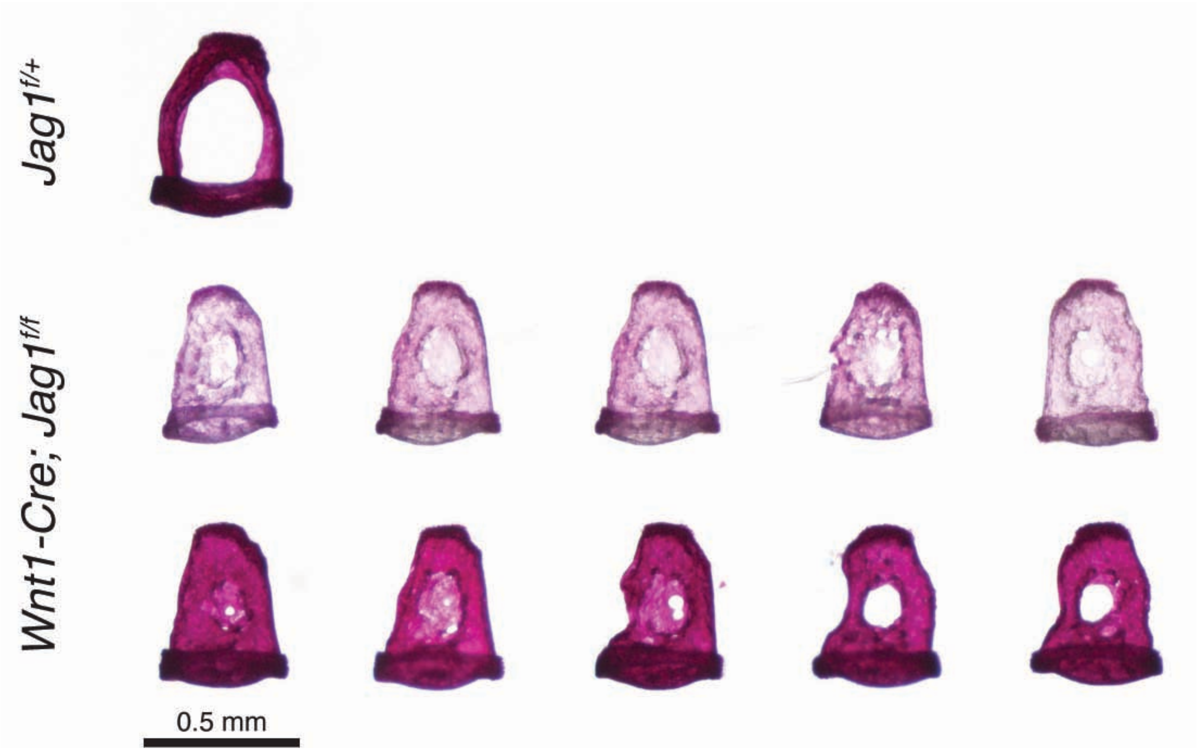

Figure S1. Complete penetrance of stapes defects in *Jag1* CKO mice.

Dissected stapes bones were stained with Alizarin Red S. *Wnt1-Cre; Jag1*$^{f/f}$ mice display a fully penetrant columellar stapes phenotype, although there is some variability in the extent of ectopic ossification within the reduced foramen.

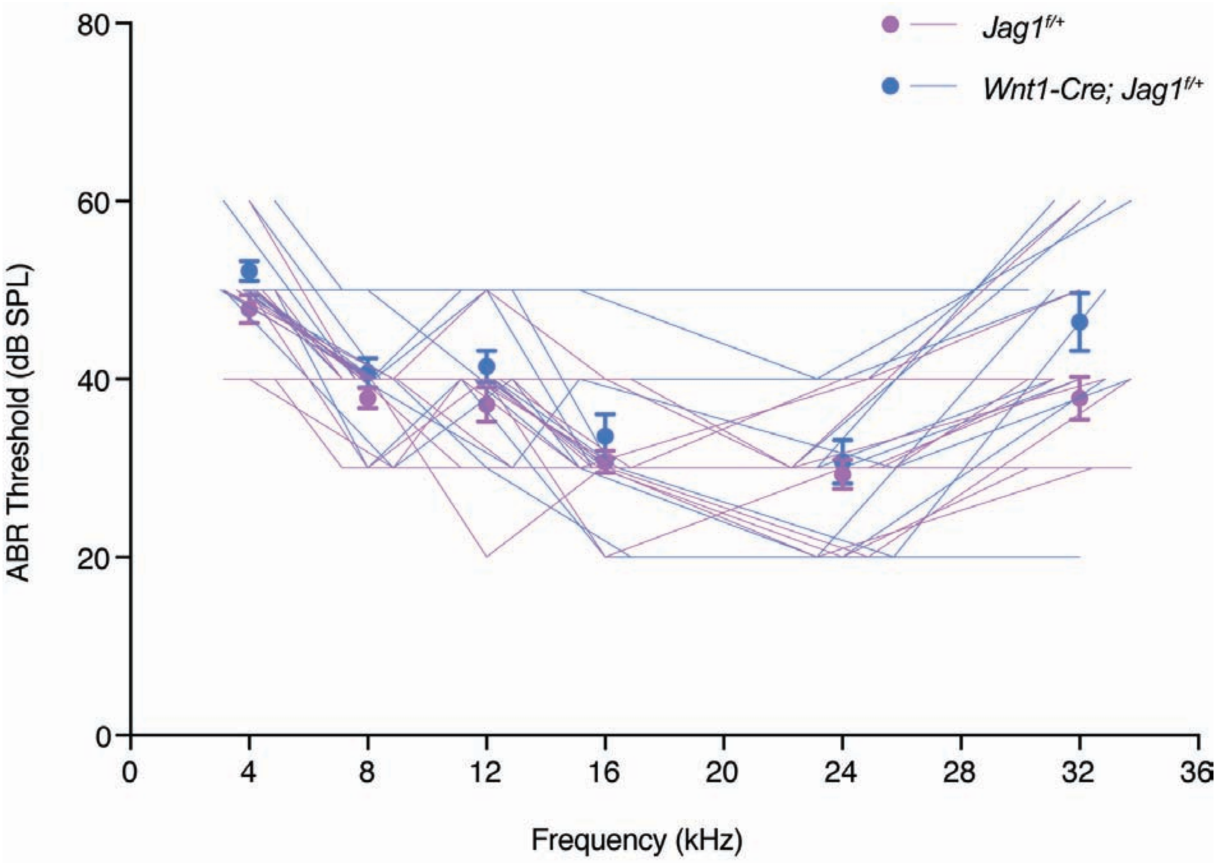

Figure S2. Normal hearing in five-week-old mice lacking one copy of *Jag1* in NCCs. At five weeks of age, *Wnt1-Cre; Jag1$^{f/+}$* (*n* = 7) and *Jag1$^{f/+}$* (*n* = 7) mice did not have significantly different hearing thresholds. Lack of difference at 4 kHz (p = 0.24), 8 kHz (p = 0.35), 12 kHz (p = 0.10), 16 kHz (p = 0.56), 24 kHz (p = 0.53), and 32 kHz (p = 0.08) was determined by two-tailed student's t-tests. Circles represent averages, and lines represent individually tested ears. Error bars represent the standard error of the mean. See also Figure 4.

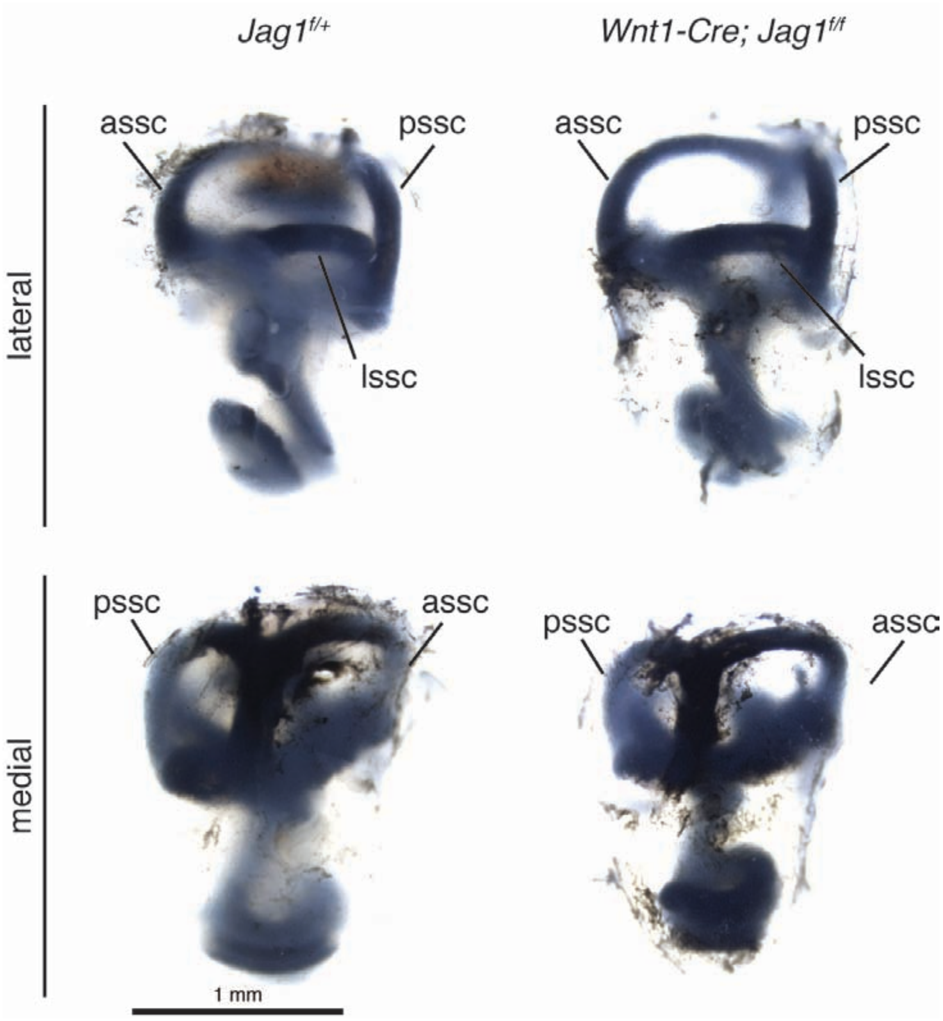

Figure S3. Normal semicircular canals in *Jag1* CKO mice.

At P0, the anterior (assc), posterior (pssc), and lateral (lssc) semicircular canals of *Jag1*$^{f/+}$ (n = 6) and *Wnt1-Cre*; *Jag1*$^{f/f}$ (n = 6) mice were not noticeably different. Canals were filled by India ink injection of dissected ears.

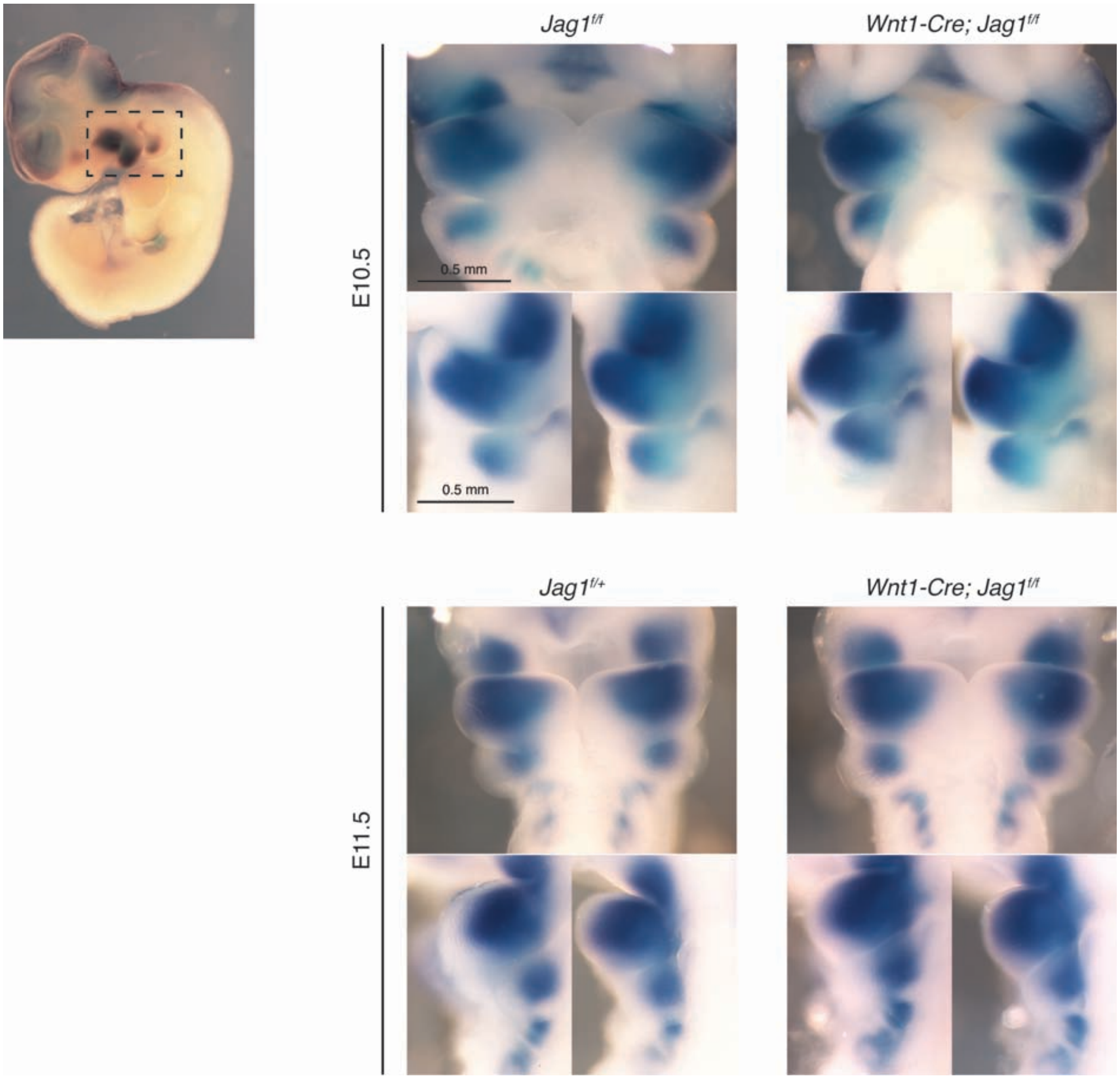

Figure S4. *Barx1* expression in conditional *Jag1* mutants. At E10.5, *Wnt1-Cre; Jag1*$^{f/f}$ (n = 4) and *Jag1*$^{f/f}$ controls (n = 4) mice did not have noticeable differences in *Barx1* expression. At E11.5, *Barx1* expression was also similar between *Wnt1-Cre; Jag1*$^{f/f}$ (n = 5) and *Jag1*$^{f/f}$ controls (n = 4). Ventral views of the pharyngeal arches are shown at the top of each panel, with lateral views of the left and right sides shown below. Anterior is to the top in each image. As a reference in the top left, a lateral view of an E10.5 wild-type embryo stained for *Barx1* is shown, with the pharyngeal arch region indicated by the dashed box. Whole-mount *in situ* hybridization was carried out as previously described [1] using the published *Barx1* probe [2].

Table S1. Results from hearing tests at the 2011 and 2014 Alagille Syndrome Alliance meetings. Unless otherwise noted, all subjects had clinical diagnosis of AGS.

Subject	Age Tested	Mutations	Ear	Tympanogram	Type of Hearing Loss	Degree of Hearing Loss
1	16	*JAG1*	R	normal	-	-
			L	normal	-	-
2[a]	9	*JAG1*	R	slightly reduced eardrum mobility with normal pressure	sens	mild
			L	normal	-	-
3	8	*JAG1*	R	negative ME pressure/ normal compliance	mixed	mild
			L	negative ME pressure/ normal compliance	mixed	mild-severe
4	7	*N.D.*	R	normal	-	-
			L	normal	cond	mild
5[b]	49	*JAG1*	R	normal	sens	normal-mild
			L	normal	mixed	mild-profound
6	5	N.D.	R	no ear drum mobility	cond	moderate
			L	no ear drum mobility	cond	moderate
7	5	N.D.	R	negative ME pressure/ normal compliance	cond	mod-normal
			L	negative ME pressure/ normal compliance	cond	mod-normal
8	6	N.D.	R	negative ME pressure/ normal compliance	cond	mild-normal
			L	normal	-	-
9	33	N.D.	R	normal	sens	mild-profound
			L	normal	sens	mild-profound
10	7	N.D.	R	normal	-	-
			L	normal	cond	normal-mild
11	3	N.D.	R	negative ME pressure/ reduced compliance	cond	unknown
			L	negative ME pressure/ normal compliance	cond	unknown
12	11	N.D.	R	normal	-	-
			L	normal	-	-
13	13	N.D.	R	normal	mixed	normal-mod
			L	normal	mixed	normal-mod
14	15	N.D.	R	normal	-	-
			L	normal	-	-
15	11	N.D.	R	normal	-	-
			L	normal	-	-
16	15	N.D.	R	normal	-	-
			L	normal	-	-
17	36	*JAG1* mosaic	R	normal	-	-
			L	normal	-	-

18	16	N.D.	R	no ear drum mobility	mixed	normal-mod
			L	slightly reduced eardrum mobility	mixed	normal-mild
19	16	N.D.	R	normal	mixed	normal-mild
			L	normal	cond	normal-mild
20	26	N.D.	R	normal	sens	normal-mild
			L	normal	sens	normal-mild
21	3	N.D.	R	no ear drum mobility	cond	normal-mild
			L	no ear drum mobility	cond	normal-mild
22	15	N.D.	R	normal	-	-
			L	normal	cond	mild-mod
23	11	N.D.	R	normal	-	-
			L	normal	-	-
24	41	N.D.	R	normal	-	-
			L	normal	-	-
25	9	*JAG1*	R	normal	-	-
			L	normal	-	-
26	32	*JAG1*	R	normal	-	-
			L	normal	-	-
27	12	*JAG1*	R	normal	cond	normal-mild
			L	normal	-	-
28	3	*JAG1*	R	negative ME pressure/ normal compliance	cond	normal-mild
			L	negative ME pressure/ normal compliance	cond	mild
29	5	N.D.	R	CNT	-	-
			L	CNT	-	-
30	3	*JAG1*	R	negative ME pressure/ normal compliance	-	-
			L	negative ME pressure/ normal compliance	-	-
31	10	*JAG1* (de novo)	R	normal	cond	normal-mild
			L	normal	-	-
32	7	*JAG1*	R	CNT	-	-
			L	CNT	-	-
33	19	N.D.	R	reduced compliance	-	-
			L	reduced compliance	-	-
34	10	*JAG1*	R	normal	cond	normal-mild
			L	normal	-	-
35	12	N.D.	R	normal	-	-
			L	normal	-	-
36	10	N.D.	R	normal	-	-
			L	normal	-	-
37	4	N.D.	R	negative ME pressure/ normal compliance	cond	mild
			L	CNT	cond	mild
38	24	N.D.	R	no ear drum mobility	cond	normal-mild
			L	no ear drum mobility	mixed	normal-mild
39	13	*JAG1* (deletion)	R	no ear drum mobility	sens	mild-mod
			L	CNT	mixed	mod-severe

40	16	*JAG1* (de novo)	R	hypercompliance	-	-
			L	normal	-	-
41	5	*JAG1*	R	reduced compliance	cond	mild
			L	normal	-	-
42	24	N.D.	R	CNT	-	-
			L	CNT	cond	normal-mild
43[c]	12	N.D.	R	no ear drum mobility	cond	normal-mild
			L	no ear drum mobility	cond	normal-mild
44[d]	23	N.D.	R	CNT	-	-
			L	CNT	-	-

cond = conductive hearing loss; CNT = could not test; L = left; mod = moderate; ME = middle ear; N.D. = not determined; R = right; sens = sensorineural hearing loss; - = within normal limits.

[a] Previous test results showed conductive hearing loss in right ear.

[b] This subject has mutations in *JAG1* but was not previously clinically diagnosed with AGS.

[c] Previous test results showed hearing at 250Hz was affected in both left and right ears.

[d] Previous test results showed conductive hearing loss in both left and right ears at 250-500Hz.

Supplementary References:

1. Hogan, B. *Manipulating the mouse embryo : a laboratory manual*, (Cold Spring Harbor Laboratory Press, Plainview, N.Y., 1994).
2. Mitsiadis, T.A. & Drouin, J. Deletion of the Pitx1 genomic locus affects mandibular tooth morphogenesis and expression of the Barx1 and Tbx1 genes. *Developmental biology* **313**, 887-896 (2008).
